# Non-discriminatory Exclusion Testing as a Tool for the Early Detection of Foot-and-Mouth Disease Incursions

**DOI:** 10.3389/fvets.2020.552670

**Published:** 2020-11-19

**Authors:** Michael Eschbaumer, Andrea Vögtlin, David J. Paton, Jamie L. Barnabei, Manuel Jose Sanchez-Vazquez, Edviges Maristela Pituco, Alejandro Mauricio Rivera, Dwane O'Brien, Charles Nfon, Emiliana Brocchi, Labib Bakkali Kassimi, David J. Lefebvre, Roberto Navarro López, Eduardo Maradei, Sergio J. Duffy, Angelika Loitsch, Kris De Clercq, Donald P. King, Stéphan Zientara, Christian Griot, Martin Beer

**Affiliations:** ^1^Institute of Diagnostic Virology, Friedrich-Loeffler-Institut, Greifswald-Insel Riems, Germany; ^2^Institute of Virology and Immunology, Sensemattstrasse, Mittelhäusern, Switzerland; ^3^Department of Infectious Diseases and Pathobiology, Vetsuisse Faculty, University of Bern, Bern, Switzerland; ^4^The Pirbright Institute, Ash Road, Woking, Surrey, United Kingdom; ^5^National Animal Vaccine and Veterinary Countermeasures Bank, Foreign Animal Disease Diagnostic Laboratory, Plum Island Animal Disease Center, Greenport, NY, United States; ^6^Centro Panamericano de Fiebre Aftosa y Salud Pública Veterinaria—PANAFTOSA, Rio de Janeiro, Brazil; ^7^Diagnostic Surveillance and Response, Australian Animal Health Laboratory, CSIRO, Australian Center for Disease Preparedness, East Geelong, VIC, Australia; ^8^National Center for Foreign Animal Disease, Canadian Food Inspection Agency, Winnipeg, MB, Canada; ^9^Istituto Zooprofilattico Sperimentale Della Lombardia e Dell'Emilia Romagna, Brescia, Italy; ^10^Animal Health Laboratory, UMR1161 Virology, INRAE, Anses, ENVA, Paris-Est Créteil University, Paris, France; ^11^Sciensano, Scientific Direction of Infectious Diseases in Animals, Service for Exotic Viruses and Particular Diseases, Brussels, Belgium; ^12^Servicio Nacional de Sanidad, Inocuidad y Calidad Agroalimentaria (SENASICA), Ciudad de México, Mexico; ^13^Private Consultants for Animal Health and Epidemiology, Buenos Aires, Argentina; ^14^Austrian Agency for Health and Food Safety, Vienna, Austria

**Keywords:** FMD, early detection, transboundary disease, exclusion testing, surveillance

## Abstract

Endemic circulation of foot-and-mouth disease (FMD) in Africa and Asia poses a continuous risk to countries in Europe, North America, and Oceania which are free from the disease. Introductions of the disease into a free region have dramatic economic impacts, especially if they are not detected at an early stage and controlled rapidly. However, farmers and veterinarians have an obvious disincentive to report clinical signs that are consistent with FMD, due to the severe consequences of raising an official suspicion, such as farm-level quarantine. One way that the risk of late detection can be mitigated is offering non-discriminatory exclusion testing schemes for differential diagnostics, wherein veterinarians can submit samples without the involvement of the competent authority and without sanctions or costs for the farmer. This review considers the benefits and limitations of this approach to improve the early detection of FMD in free countries and gives an overview of the FMD testing schemes currently in use in selected countries in Europe and the Americas as well as in Australia.

## Introduction

Foot-and-mouth disease (FMD) is a highly contagious vesicular disease of cloven-hoofed animals caused by an aphthovirus in the family *Picornaviridae*. The main clinical signs of FMD are lesions on the tongue, oral mucosa and nasal planum, on the teats and in the interdigital spaces and coronary bands of the feet. Except in very young stock, mortality is generally low, but the reduced productivity and the loss of draft power cause significant economic hardship and food insecurity in endemic areas, which are exacerbated by the costs of control measures, and the forfeiture of trade revenue (1).

**Figure 1:**
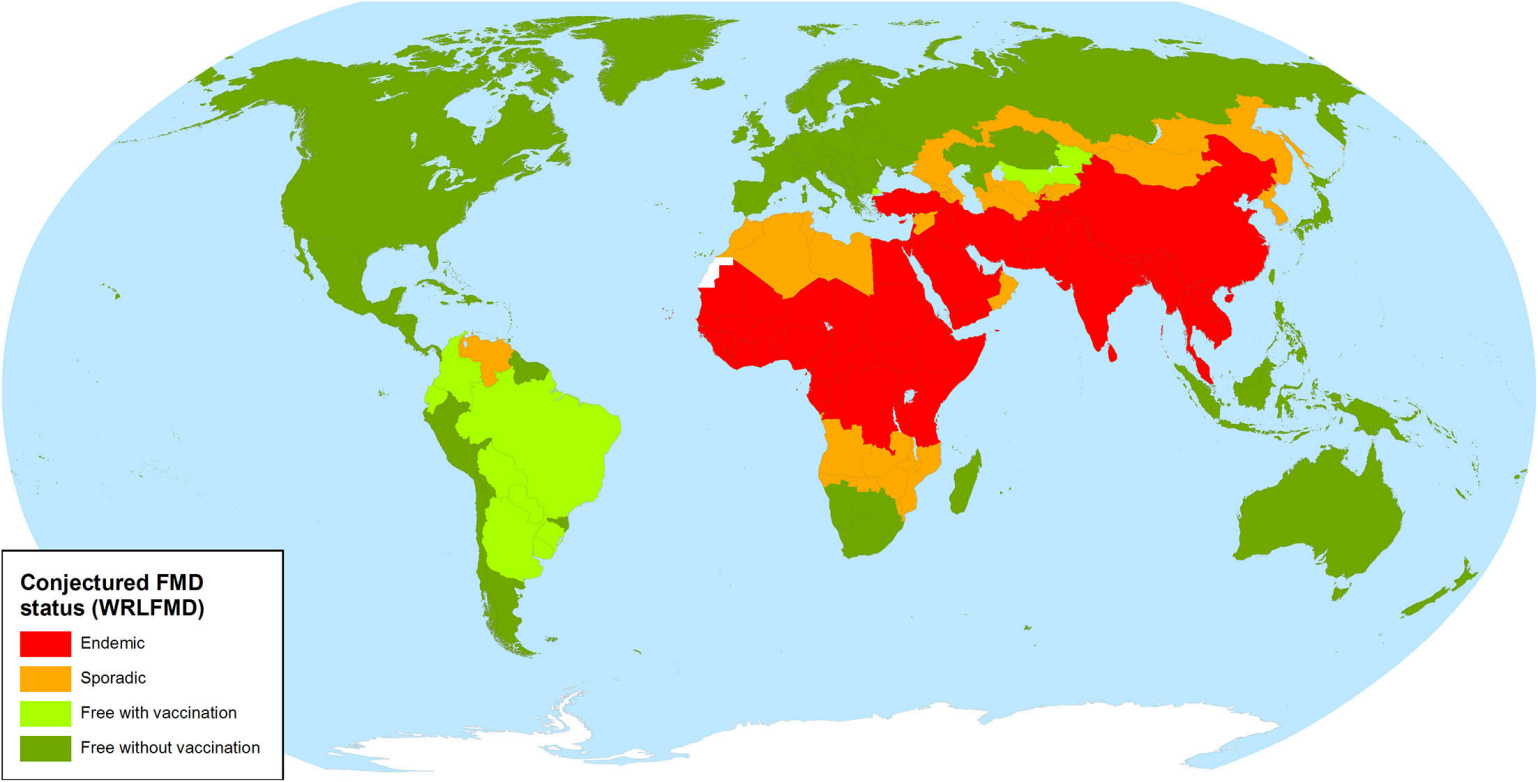
FMD status map of the World Reference Laboratory for FMD (4).

FMDV has not occurred in Europe, North America and Oceania for almost 10 years; the last FMD outbreak in any of these regions was in Bulgaria during 2011 (2), while North America and Oceania have been free for much longer. FMD was eradicated in Australia, the United States, Canada and Mexico in 1872, 1929, 1952, and 1953, respectively (3), and has never been reported from New Zealand. However, it is still endemic in Africa and Asia (see also the map in Figure 1) (4), and there is always a serious risk of the virus being reintroduced, particularly through the illegal import of animal products. Introductions of FMD into free regions have dramatic social and economic impacts, especially if they are not detected and controlled rapidly (1).

## The Case for Non-Discriminatory FMD Exclusion Testing

It has often been argued by proponents of pen-side testing that the time from sample submission to the return of results from the diagnostic laboratory is a significant obstacle to the rapid detection of an FMD incursion (5, 6). While this may be the case in areas with insufficient transportation and laboratory infrastructure, the turnaround time in countries with highly industrialized agriculture and sophisticated veterinary services is typically short. For “hot” initial suspicions, samples are sent to official laboratories by courier or government vehicle and tested immediately upon arrival, with results usually returned within 24 h (7–9).

**Figure 2:**
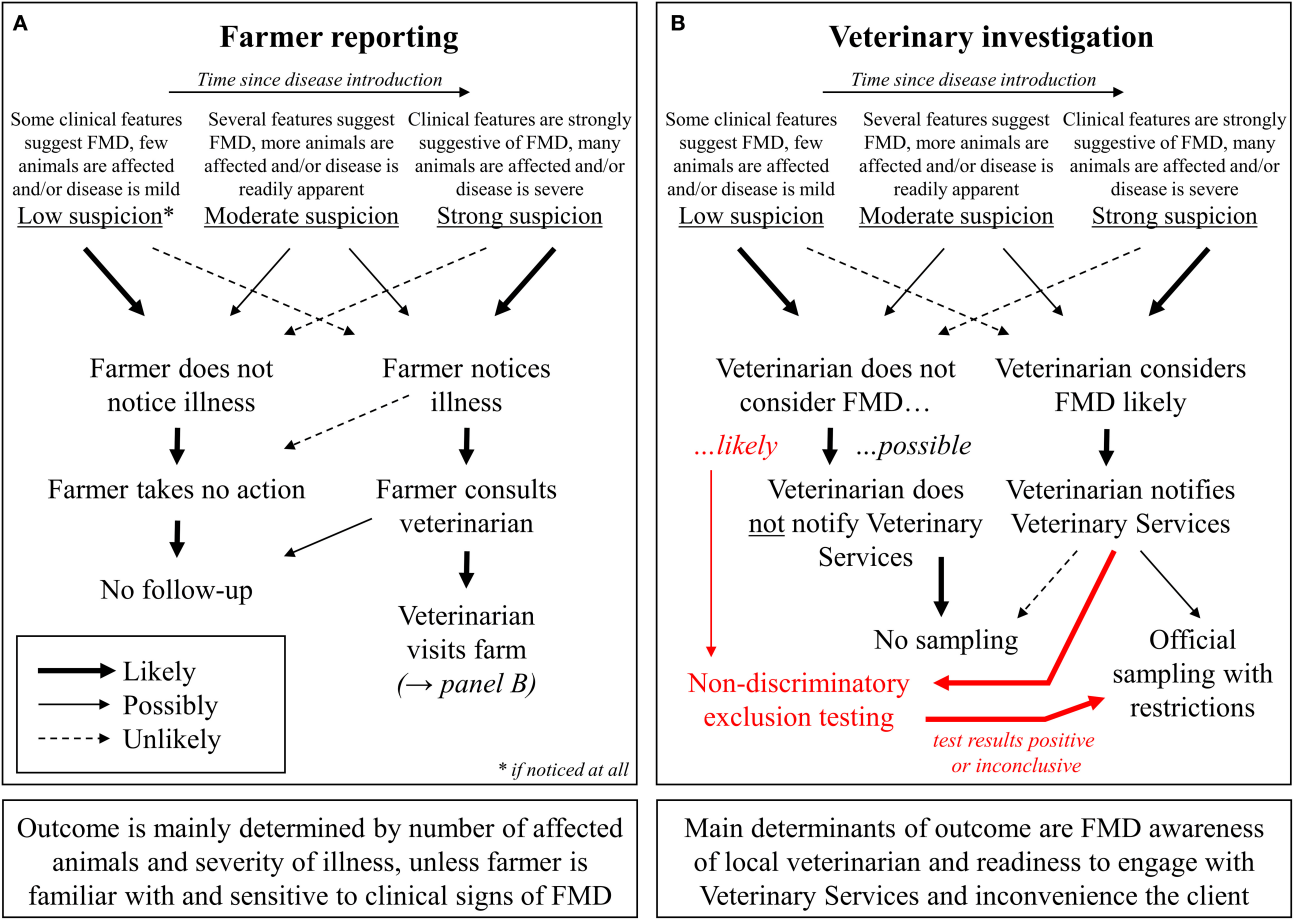
Potential roles of farmers **(A)**, as well as veterinary practitioners and official veterinarians **(B)**, in FMD reporting.

By contrast, much longer periods of time can pass between infection, the first occurrence of clinical signs, their recognition by the farmer, and finally the submission of samples for laboratory testing. The first obstacle here is the recognition of clinical signs and the realization that they may be an indication of a larger issue that requires veterinary attention (see Figure 2A). The severity and within-herd prevalence as well as the risk of onward transmission are related to the time that has elapsed since the introduction of FMDV into the herd. Therefore, it is essential that reporting occurs as early as possible, even at a mild incipient suspicion of disease, rather than waiting for a high level of morbidity. These factors are critical elements for limiting the size of outbreaks after a disease incursion into an FMD-free country. Laboratory testing will not occur unless a problem is reported to an official or private veterinarian in the first place. Accordingly, efforts to improve reporting by livestock owners (e.g., awareness programs, streamlined reporting procedures, or the availability of telephone “hotlines”) should be considered. But, even if farmers come to realize that there is a problem, some may decide not to consult a veterinarian because of cost implications, a lack of trust in animal health authorities, or the fear of consequences for themselves or their animals (10–13).

An example of this is the large series of FMD outbreaks that originated in the United Kingdom (UK) in 2001 and ultimately affected four countries in Europe, resulting in the culling of over 6 million animals as well as economic losses of 8 billion Euros (1). After the country had been free of FMD for 34 years, a serotype O strain of ultimately unknown, but most likely East Asian origin was introduced into a pig fattening unit in northern England by illegal feeding of untreated catering waste (14). This was not realized until weeks later, when clinical signs were detected in pigs that became infected at an abattoir that had received animals from the index herd. At this point, the disease had already spread widely, and it took over 6 months to bring the outbreak under control (15). Sheep played a large role in the early dissemination of the virus, and it was later determined that sheep farmers had also noticed lameness in their animals but did not seek veterinary advice (15). While the pig farmer was found guilty of deliberately hiding the disease in his animals (16), it appears that the sheep farmers were genuinely unaware of the implications of their observations.

In areas where a particular disease has not occurred in decades, most farmers and practitioners are unfamiliar with its clinical manifestations (11). There are a wide variety of conditions (both infectious and non-infectious) that can cause clinical signs that are similar to those of FMD (e.g., oral and/or pedal lesions or lameness) (17), and particularly in countries with routine outbreaks of clinically indistinguishable vesicular diseases, there can be a tendency to think that observed signs are caused by anything but FMD (11). For example, the only outbreak of FMD in Canada in 1951 was initially misdiagnosed as vesicular stomatitis, and samples for laboratory diagnosis were first submitted almost 3 months after the first observation of clinical signs (18). Increased incidence of porcine dermatitis and nephropathy syndrome (PDNS) in the UK likely contributed to the delayed detection of classical swine fever (CSF) in 2000. After a false diagnosis of PDNS had been made in one of the holdings first affected, CSF was not suspected for a further 2 weeks, until losses had accelerated steeply (19). In a more recent example from Germany, it took more than 3 months until a highly virulent strain of bovine viral diarrhea virus was identified as the cause of severe disease in several cattle holdings (20). From the beginning of the outbreak, clinical signs noted by farmers—and veterinarians—on the affected premises had included fever and tongue lesions, but at no point were these animals tested for FMD.

In cases where FMD cannot be ruled out based on the clinical picture alone, samples must be submitted for laboratory examination. However, if the collection and examination of these samples is contingent on the declaration of a formal suspicion of FMD, this can set the bar for a submission very high. Except in the most obvious cases, some veterinary practitioners may be reluctant to raise an FMD suspicion because of the consequences that are precipitated by its formal declaration, regardless of whether the suspicion is eventually confirmed by laboratory diagnosis (see also Table 1). In the European Union (EU), national animal health laws reflect the provisions of articles 4–9 of the “FMD Directive” 2003/85/EC, requiring a quarantine of the suspect holding and other holdings around it, a movement ban for all animals in a large area around the suspect holding, and even the preemptive killing of animals of susceptible species if this is deemed necessary by the competent authority (21). Similar regulations are in place in other FMD-free countries (9, 22, 23).

**Table 1:** Benefits, limitations, and risks associated with non-discriminatory exclusion testing schemes for FMD.

**Benefits**	**Limitations and Challenges**	**Risks**
− Allows veterinary practitioners to consider FMD as a differential diagnosis without fear of negative consequences for the farmer or themselves − Testing of low-risk exclusion samples at regional laboratories creates and maintains surge capacity for outbreak response	− Requires outreach to farmers and veterinarians to promote participation − Farmers still need to notice clinical signs and seek veterinary attention first − Requires clear definition of exclusion cases vs. notifiable suspicions − Veterinary services must be kept informed and quickly consulted if problems arise during exclusion testing	− If exclusion testing is used instead of immediate notification in serious cases, an appropriate outbreak response may be delayed − False-negative results can delay the recognition of an FMD incursion − May not be acceptable to trading partners

Unsurprisingly, official veterinarians may be viewed by practitioners and farmers in an adversarial role. Even though they are required to do so by law in most jurisdictions, practitioners can be reluctant to report diseases that could result in regulatory action and may be afraid of alienating their clients if they raise suspicions that turn out to be false alarms (24) (see also Table 1). This is not entirely unreasonable, because the prior probability of FMD being the cause of suspicious lesions is very low in areas that are free of the disease (15). Nevertheless, if the reporting of suspected foreign animal diseases is only the very last resort, it creates a serious obstacle for their timely detection and control (25).

Notwithstanding the critical importance of implementing rapid control measures in the event of a credible disease suspicion, a possible approach to increase FMD surveillance is to allow veterinary practitioners to submit samples for an exclusion of FMD without restrictions for the farm (with or without the direct involvement of the competent authority; see Figure 2B). The laboratory costs for such non-discriminatory exclusion testing should be covered by communal animal health funds or the government, because increased testing helps to protect the entire agricultural sector from disaster, and there should not be any financial barriers to participation for individual farmers (11, 24). In addition to excluding FMDV infection, possible differential diagnoses (both endemic and exotic) can be covered by the laboratory investigation, ideally at no additional charge. This added value will further encourage farmers and their veterinarians to participate in the exclusion testing system (see also Table 1). Official testing for notifiable diseases such as FMD is often unsatisfactory for farmers because it does not return a useful diagnostic result once the target diseases have been ruled out.

Where non-discriminatory exclusion testing schemes for FMD are already available (e.g., Switzerland and Germany) (8, 26), educating and encouraging practitioners to make use of these programs is an important part of community outreach activities of the veterinary services (see also Table 1). In addition to the classical signs of FMD, exclusion testing can also be warranted for non-specific health problems in a herd. For example, German animal health law has recently been revised to require cattle farmers who observe an increased incidence of febrile illness, a significant reduction in milk yield or increased mortality in young stock to consult a veterinarian to rule out FMDV infection (cf. Section 2a of the *Ordinance on Protection against FMD*^1^). Similar rules for swine holdings have been in place since 1999. At the same time, it must be made very clear to practitioners that the exclusion testing option is not to be used if they actually *suspect* FMD! If critical samples are submitted for exclusion testing only, this can lead to delays in disease confirmation and containment because they are not considered a high priority for delivery to and processing at the diagnostic laboratory.

In the EU, while samples from suspect cases must always be sent to the designated national reference laboratory (NRL), exclusion testing by real-time RT-PCR (RT-qPCR) can be done in any laboratory designated by the competent authority (cf. Annex XV No. 13 of the “FMD Directive”) (21). Suitable samples for RT-qPCR tests are lesion material, if available, as well as saliva and serum. In FMD-free countries, laboratories accepting these samples do not need to operate under high-containment conditions, but they are obliged to follow procedures that ensure that the spread of possible FMDV present in the sample material is effectively prevented (27). This includes, among other things, the spatial and organizational separation of areas in which FMD exclusion testing is carried out, the use of Class II microbiological safety cabinets for the processing of samples that have not yet been chemically or physically inactivated, and strict hygiene management for work surfaces, equipment, laboratory waste, and personal protective equipment. All laboratories that do exclusion testing must operate to the highest diagnostic standards. In addition to ISO accreditation, this should include successful participation in FMD diagnostic proficiency tests periodically administered by the NRL. The NRL should be kept informed of any exclusion testing performed by these other laboratories.

If the results of an exclusion test at another laboratory are not clearly negative, the samples must be immediately forwarded to the NRL for clarification (see Figure 2B). Under this scenario, fresh sampling of the animals may be required to ensure the provenance of samples and to eliminate any possibility of accidental cross-contamination at the first laboratory. When sending these samples, the transport regulations for category B biological substances (UN3373) must be observed. At the same time, the veterinary authority responsible for the holding where the samples have been taken must be notified of the situation and it is likely that the farm would be placed under formal suspicion until results from the NRL are known. As long as the first-line tests employed have high specificity, at this point, it is no longer a question of exclusion testing or elimination of differential diagnoses but may already rise to the level of a formal suspicion of FMD. If a formal suspicion of FMD is declared, testing to confirm or clear that suspicion can only be carried out at the designated NRL (cf. Annex XV No. 5 and 13 of the “FMD Directive”) where it will be treated with high priority.

Overall, when a non-discriminatory exclusion testing scheme is implemented in a country, it is advisable for the veterinary authorities to create guidelines for practitioners. This should include a decision tree to determine whether or not a case is likely to be FMD (based on the clinico-pathological presentation and the epidemiological context), standard procedures for sample collection and submission, and any follow-up actions or reporting requirements. The veterinary authorities need to devise a strategy for the ongoing training of practitioners to make sure they know how to recognize FMD and the conditions for applying non-discriminatory exclusion testing. Moreover, an active communication channel from the veterinary authorities to practitioners needs to be established to be able to timely disseminate relevant information. For example, this can be used to update stakeholders about the regional risk of FMD according to its presence in neighboring countries, as this information will influence the decision to consider FMD as a likely or unlikely differential in a given case.

## FMD Exclusion Testing in Practice

### Europe

Switzerland introduced non-discriminatory exclusion testing in 2011. These examinations are carried out at two central government laboratories and cover not only FMD but also African swine fever (ASF), CSF, avian influenza (AI), and Newcastle disease. Samples are submitted by veterinary practitioners and pathologists and include mostly sera, lesions or swabs; in addition, EDTA blood is requested to test for bluetongue (BT) virus as a potential differential diagnosis. Since the costs are covered by the government through the Federal Food Safety and Veterinary Office, the exclusion examinations are free of charge for the senders. The program is considered a success both by the veterinary services and by practitioners, and it has led to a marked increase in the number of samples tested for foreign animal diseases (23). From January 2012 to February 2020, no “hot” suspicions were declared, but 101 FMD exclusion tests were performed. Nevertheless, considering the number of cattle, sheep, goats and pigs in Switzerland (1.5 million, 340 thousand, 80 thousand and 1.3 million, respectively), it would be desirable to receive even more samples for exclusion testing. In a large population of animals, there inevitably is some “background noise” of oral lesions or lameness, due to endemic infectious diseases such as contagious pustular dermatitis (orf) or non-infectious causes such as chemical burns. For example, a recent field study in sheep (28) found oral lesions in 1% of animals. Even if most of these can be determined not to be FMD by other means, a large number of cases will remain that warrant laboratory diagnosis.

Belgium is using a risk-based “increased vigilance” scheme. Among others, this increased vigilance is currently applied to BT, ASF, and AI. The criteria that trigger exclusion diagnostics in the absence of a clinical suspicion are very different for each disease: for BT, it is importation of ruminants from risk areas with serotypes other than serotype 8; for ASF, it is 2 or more pigs on a farm with symptoms of general disease; for AI, it is abnormal production parameters (e.g., increased mortality). Within the “increased vigilance” scheme, the costs for sampling (or necropsy) are borne by the owner of the animals, whereas laboratory analysis is paid for by the Federal Agency for the Safety of the Food Chain (FASFC). As a result of the aforementioned risk analysis, there currently is no organized system for exclusion testing for FMD and other vesicular diseases, even though these tests are available. Every decision for exclusion testing for vesicular diseases is made on an *ad-hoc* basis. Reasons for FMD exclusion testing can be. e.g., a herd problem of unknown etiology, a (presumably false) positive result in an antibody test for export certification, a lesion at the mouth or foot observed at necropsy or irregularities with animal identification or documentation of origin. In the latter case, the exclusion testing must be paid for by the owner, otherwise it is paid for by the FASFC since laboratory analysis is then done in the context of a—perhaps not (yet) formally expressed—suspicion. On average, there are ≤ 2 clinical suspicions and ≤10 exclusion diagnostics for vesicular diseases per year in Belgium.

Austria introduced a non-discriminatory exclusion testing scheme in 2014, which was revised in 2019^2^. It consists of five stages (see Table 2), whereby stage II (exclusion testing) is divided into two sub-stages A and B. An earlier contingency plan established in 2000 had only included provisions for suspected holdings and holdings with an outbreak (29), similar to stages III, and IV of the new scheme.

**Table 2:** Stages of the Austrian FMD testing scheme.

**Stage**	**I**	**II A**	**II B**	**III**	**IV**
**Type of investigation**	**Differential diagnosis**	**Exclusion testing**		**Suspicion**	**Outbreak**
Respondent	Any veterinarian		Official veterinarian		
Laboratory	Any	NRL	NRL	NRL	NRL
Costs covered by ministry	No	Yes	Yes	Yes	Yes
Quarantine of the farm	No	No	No	Yes	Yes

In routine cases (stage I), any diagnostic laboratory can test for FMDV as a differential diagnosis, but all exclusion testing (stage II), and investigations of FMD suspicions or outbreaks take place at the Austrian NRL. Any veterinarian can submit samples for FMD exclusion testing directly to the NRL or can notify veterinary services. This notification is mandatory if the veterinarian actually suspects FMD! Upon notification, the official veterinarian visits the farm and determines the level of suspicion. If there is only a weak indication of the disease, samples will be submitted for exclusion testing (stage II.B) and no restrictions are imposed on the farm. If the suspicion of FMD (or any other notifiable disease) is confirmed by the official veterinarian, stage III (or IV, depending on severity) will be declared and the holding will be quarantined.

Exclusion testing in Austria is free of charge for the farmer, since the central competent authority covers the costs of the testing. However, if the farmer wants a test for a non-notifiable disease from the same sample material (in addition to the exclusion testing), they have to cover the costs of this additional testing. Although non-discriminatory exclusion testing has been offered in Austria since 2014, not many samples have been submitted to the NRL. There are <5 cases of exclusion testing for vesicular diseases per year in Austria, for a susceptible population of 1.9 million cattle, 0.5 million sheep, 0.1 million goats, and 3 million pigs.

In Germany, the regional veterinary diagnostic laboratories of all federal states (with the exception of the city states, which only have negligible numbers of livestock), have offered exclusion testing for FMD since 2014, following the earlier implementation of distributed non-discriminatory testing for CSF, AI, and ASF. Private laboratories do not test for FMDV. The participating regional laboratories are enrolled in proficiency tests conducted by the NRLs (30, 31) and will forward positive or inconclusive samples to the NRL for confirmation. Due to the sovereignty of the individual states in matters of animal health, the terms and conditions under which the program is conducted are variable, as is the acceptance among practitioners and the number of samples submitted (32). From 2014 to 2016, the number of samples tested for FMDV RNA across all laboratories increased from 281 to 729 (32) without the concurrent emergence of another vesicular disease such as Senecavirus A (SVA) infection (33), as has happened in the United States (see below). This shows that the concept of FMD exclusion testing is gaining acceptance. But, similar to the situation in Switzerland, the number of tests is still far too low compared to the population of susceptible animals in Germany (2016: 12.3 million heads of cattle, 2 million small ruminants and 28 million pigs), and has to be further increased.

### Australia

Similar to the situation in Germany, each of the states and territories that make up the Australian federation operate under different animal health legislation and the Chief Veterinary Officer (CVO) of the relevant state determines if any legal restrictions should apply. Legally binding on-farm restrictions are not mandated whenever an FMD laboratory test is conducted. This allows for exclusion testing to be carried out where FMD is not thought to be a probable differential diagnosis however clinical signs such as lameness or salivation are present. This approach applies across all nationally notifiable diseases and aims to encourage exclusion testing for all livestock species. In the 3 years between January 2017 and December 2019, 215 exclusions for vesicular diseases were carried out in Australia (Table 3). All exclusion testing for nationally notifiable livestock diseases is funded by the commonwealth government and testing is carried out by the Australian Center for Disease Preparedness in Geelong. Detailed case reports on FMD exclusions are frequently published in the Australian Animal Health Surveillance Quarterly (34) (see examples in Table 3).

**Table 3:** FMD exclusion investigations done in Australia between 2017 and 2019 as reported in the Australian Animal Health Surveillance Quarterly (AHSQ).

**Reported in AHSQ issue**	**Cattle**	**Sheep**	**Camel**	**Alpaca**	**Goat**	**Pig**	**Buffalo**	**FMD exclusion reports**
2017–Vol 22/1	8	2	0	0	0	0	0	
2017–Vol 22/2	9	6	0	0	0	1	0	p. 40
2017–Vol 22/3	8	5	0	0	0	2	0	p. 20, 33
2017–Vol 22/4	9	8	0	0	0	1	1	p. 18–19. 25
2018–Vol 23/1	10	5	0	0	0	3	0	p. 44
2018–Vol 23/2	20	6	1	0	2	0	0	p. 42
2018–Vol 23/3	10	3	0	0	0	1	0	p. 38
2018–Vol 23/4	9	2	0	0	0	2	0	
2019–Vol 24/1	13	2	0	1	1	0	0	p. 20
2019–Vol 24/2	13	4	0	0	1	1	0	p. 19
2019–Vol 24/3	19	7	0	1	0	0	0	p. 17–18, 19
2019–Vol 24/4	13	4	1	0	0	0	0	
Total (2017–2019)	141	54	2	2	4	11	1	

In addition to the laboratory testing being funded by the national government, Australia also has in place a National Significant Disease Investigation Program (SDI) (35). This program provides subsidized veterinary services and diagnostic testing up to the value of AU$1100 to investigate disease events (36). This program aims to encourage non-government veterinarians to investigate the cause of a significant disease event even when the commercial value of the animal is less than the veterinary services required. Although not specifically targeted at vesicular diseases, the SDI program often results in government and non-government veterinarians working together to solve animal health problems.

## Systems in Use in Countries that do not Allow Non-Discriminatory Exclusion Testing

### Europe

The UK has adopted a binary perspective for initial report cases of FMD in the country—i.e., either (i) there is credible suspicion of disease and on-farm restrictions are adopted until laboratory results are generated or evidence for disease freedom can be provided from other sources, or (ii) based on the clinical and epidemiological context, the animals are not considered to be infected with FMDV and no laboratory testing is necessary or even desirable. This is achieved by a two-tiered alert system (available 24 h a day with veterinary epidemiology support): in stage 1, where restrictions are immediately placed on the premises (by law), clinical suspicion of FMD leads to a visit of an official veterinarian, who undertakes clinical investigation and assesses whether samples are to be taken. If the veterinary visit cannot rule out FMD, stage 2 involves sample collection, laboratory testing and implementation of full restrictions on the farm as well as the need for area restrictions (considered by the competent authority) until a negative test result is returned.

Similar to the UK, France does not employ non-discriminatory exclusion testing for FMD, but its national reference laboratory also has veterinarians and epidemiologists available by phone 24 h a day. Veterinary practitioners who encounter alarming clinical signs can call in and describe and electronically transmit their observations with photos. Taking into account the risk profile of the holding, the epidemiological situation and the clinical signs, the central team will decide if the situation requires laboratory testing or if FMD can be ruled out without it. This relieves the veterinary practitioner of any responsibility for that decision and lowers the bar for reporting possibly suspect cases. If testing is deemed necessary (usually 1–2 cases per year for a susceptible population of 20 million cattle, 9 million sheep and 7 million pigs), the holding will be quarantined, and the samples will be forwarded to the NRL with high priority and tested immediately at any time.

Italy does not offer exclusion testing for FMD. In case of an FMD suspicion, private veterinarians must inform the veterinary services, which will then visit the suspected herd. The cost of laboratory testing for suspect cases is covered by national funds from the Ministry of Health. Formal FMD suspicions were last declared in 2015 and 2016 (2 each), for a susceptible population of 5.6 million cattle, 0.4 million buffalo, 6.2 million sheep, 1 million goats and 8.7 million pigs. Currently, virological testing for FMD in Italy is only done at the NRL, which is located at one of the ten *Istituti zooprofilattici sperimentali* (IZS) of the national animal health and food safety network. However, the IZS network is prepared to carry out post-vaccination FMD serology, and it is planned to extend the existing proficiency testing for the serological assays to include FMDV RT-qPCR. This will build up distributed diagnostic capability that will be very useful if there is an FMD outbreak. If low-risk submissions (e.g., from holdings without clinical signs) can be tested at regional laboratories, this frees up testing capacity for critical samples at the NRL during an outbreak and can decrease sample turnaround time in large countries.

### North America

In Canada, based on the experience of the 1951 outbreak (18), all suspicions of vesicular disease in Canada are considered to be FMD until proven otherwise and any suspicion must be reported to the Canadian Food Inspection Agency (CFIA) (37). Following that notification, the local CFIA veterinarian visits the premises. Based on the clinical signs and in consultation with CFIA foreign animal disease specialists, the level of risk is determined, commensurate restrictions are placed on the premises and samples are collected for laboratory testing at the National Center for Foreign Animal Disease. Samples from animals with clinical signs, morbidity, epidemiological data or other factors that indicate a high likelihood of FMD are submitted as “high risk.” For cases where the risk of FMD is low but still warrants laboratory testing, the samples are submitted as “confirmatory negative.” Even when the risk is negligible, samples can be sent for laboratory testing under the category of “disease investigation.”

In the United States, the Department of Agriculture's Animal and Plant Health Inspection Service (APHIS) is the competent authority for the detection of a foreign/transboundary animal disease (FAD/TAD). Samples for suspect vesicular diseases in livestock are sent to an approved laboratory within the National Animal Health Laboratory Network (NAHLN), a network of more than 60 state, university-associated, and federal laboratories across the country that provide both active and passive surveillance, as well as surge capacity support during an outbreak (38). Duplicate samples are also sent to the National Veterinary Services Laboratories (NVSL) Foreign Animal Disease Diagnostic Laboratory (FADDL) on Plum Island for confirmatory testing to rule out FMD, as handling live FMDV is currently prohibited on the U.S. mainland^3^. All suspect FAD investigations (FADIs) require formal notification within the National Response Framework. All FADIs begin with notification to the state animal health official (SAHO) and the federal Area Veterinarian in Charge (AVIC), at which time a veterinarian trained in FAD sample collection by FADDL, known as a Foreign Animal Disease Diagnostician (FADD), is dispatched to the location. The FADD collects duplicate samples to send to NVSL and a NAHLN laboratory proficiency tested to run the same FMDV PCR assays as FADDL.

Testing results and reporting will be expedited based on the assigned priority level. The FADD, SAHO, and AVIC will agree on a priority level based on risk and on-site epidemiology of the suspect vesicular case. Four priority levels exist: (i) Priority 3, low suspicion for/unlikely to be an FAD, but cannot be distinguished from an endemic condition. (ii) Priority 2, indication this is possibly an FAD, and cannot be distinguished from an endemic condition. Rapid laboratory confirmation is required; (iii) Priority 1, prompt laboratory confirmation is required because there is a high suspicion for an FAD; and finally (iv) Priority A, which also requires prompt laboratory confirmation and is used in situations where animals in commerce are held pending results for FAD testing (39). Testing is conducted at no cost to the customer.

In some situations, testing for endemic diseases that are clinically indistinguishable from FMD, such as SVA infection (33), may also be performed by NVSL FADDL or the NAHLN. The recent emergence of SVA in North America has led to a dramatic increase in costly official disease notifications in Canada and the USA (40). Prior to 2016, FADDL saw an average of 150 FADIs annually, but in 2017, more than 1,300 accessions were received. Through a shared testing program, 343 of 1,314 total cases in 2018 were tested by the NAHLN only and not by FADDL. In 2019, ~1,542 accessions were received; however, 687 of those were tested only at a NAHLN laboratory.

In Mexico, a specific surveillance program for vesicular diseases of animals—the binational Mexico-United States Commission for the Prevention of FMD and Other Exotic Diseases of Animals (CPA)—has been in place since 1954. Any suspect vesicular disease must be immediately reported. There is an entire program dedicated to promoting such notifications through courses, newsletters, and social networks. All suspect cases are attended to in <24 h, on any day of the year, and are handled exclusively by CPA personnel, who have the necessary equipment and means to respond to any notification of a suspect foreign animal disease. Diagnosis occurs at a single high-security laboratory and all investigations are paid for by the CPA. In 2019, 2,621 samples from 103 cases were tested for FMDV, 702/41 of which were cattle, 1,029/35 goats, 685/20 sheep, 85/4 swine, and 120/3 wildlife.

### South America

In South America, the epidemiological context and the risk perception is different than in Europe and North America. After its introduction to South America in the 19th century, FMD quickly became endemic in the large cattle population. Only through a tremendous and sustained collaborative effort over several decades has the continent now come close to eradication of the disease (41). While all of South America, apart from Venezuela and a small zone in north-eastern Colombia, is recognized as FMD-free (with or without vaccination) by the OIE (42), the fear of this disease and its economic consequences is still very present among the governments and producers in the region. Thus, there is a strong pressure to deal with any suspicion of vesicular disease as if it were FMD. Notably, vesicular stomatitis virus (VSV) is present in the region, particularly in Colombia and Ecuador (43–45). It has a pathological presentation in ruminants and pigs that is indistinguishable from FMD and laboratory testing is required to rule it out. Accordingly, in the presence of suspected vesicular disease, FMD should always be ruled out together with VSV and for swine, it is advisable to include SVA as well.

An informal opinion poll of current and former CVOs and NRL directors in South America conducted for this review revealed strong disapproval of making non-discriminatory exclusion testing available as an option for private veterinarians. It was seen as too difficult to define what constitutes “clear” or “less clear” suspicions of FMD, and even more difficult to explain such a difference to farmers and private veterinarians. There also was concern that exclusion testing may allow farmers or practitioners to intentionally delay the notification of exotic animal diseases, ultimately resulting in a failure to implement appropriate control measures in a timely manner. In addition, an epidemiological investigation by the veterinary services is seen as essential for vesicular disease suspicions because negative laboratory results may be obtained from infected herds due to inappropriate sample collection, handling or analysis.

In 2018, the 13 countries that are part of the South American Commission for the Fight Against FMD (COSALFA) reviewed the way FMD suspicions are addressed (45). It was agreed that any suspicion of vesicular disease needs to be responded to by the official veterinary services which should proceed, in the first place, with an official visit to the farm. On site, the official veterinarian will decide, based on the epidemiological investigation and the clinico-pathological presentation, whether it is a so-called “well-founded suspicion.” Well-founded suspicions should always lead to laboratory testing at the NRL and until FMD is ruled out, the farm is quarantined, and movement restrictions are applied. On the contrary, if the official veterinarian rules out FMD during the site visit the case may be closed without laboratory testing, similar to the approach taken by the UK and France.

There are, however, exceptions to this procedure. For example, when vesicular lesions are found in pigs at an abattoir in Brazil, slaughter may proceed normally if the batch is accompanied by official documentation from the veterinary services indicating that the farm of origin has been investigated for FMD with a negative result within the last 30 days (46). This is used by commercial pig farms with high biosecurity standards that are located in recognized FMD-free areas where SVA is known to be present.

In 2018, the last year for which collated data are available from the Pan American Foot-and-Mouth Disease Center (PANAFTOSA) (43), a total of 1,976 suspicions of vesicular disease were reported in 12 countries, together representing over 365 million heads of cattle and buffalo. In only 7 cases was FMDV infection confirmed by laboratory diagnosis (see Table 4).

**Table 4:** Vesicular disease notifications, laboratory diagnoses, and number of cattle and buffalo in South America in 2018.

			Positive diagnosis^*^			**Negative laboratory diagnosis**			**Number of cattle and buffalo**	
**Country**	**Notifications of vesicular disease^a^**	**“Well-founded”**	**FMDV**	**VSV**	**SVA**	**FMDV**	**VSV**	**SVA**	**Heads^b^**	**Farms^c^**
Argentina	5	0	–	–	–	5	–	–	55,546,342	205,655
Bolivia	23	0	–	–	–	23	–	–	9,092,286	183,702
Brazil	775	334	–	4	21	775	771	602	218,004,131	2,454,550
Chile	15	15	–	–	–	15	15	–	3,719,507	125,402
Colombia	428	428	7 (1)	241	18	161	–	–	27,590,935	627,239
Ecuador	575	575	–	308 (5)	–	570	114	–	4,313,264	271,590
Guyana	23	0	–	–	–	–	–	–	260,673	4,024
Panama	24	0	–	8 (16)	–	16	2	–	1,521,500	43,948
Paraguay	10	10	–	–	–	10	10	–	13,500,965	145,025
Peru	45	45	–	36 (32)	–	45	13	–	5,156,044	881,920
Uruguay	10	0	–	–	–	10	–	–	11,435,655	40,576
Venezuela	43	43	–	9 (11)	–	2	2	–	15,454,847	108,211

a *A farm with one or more susceptible animals with pertinent clinical signs*.

b *More than 95% of these are cattle*.

c *More than 98% of farms have only cattle*.

* *Either by laboratory testing or by clinico-epidemiological investigation (numbers in parentheses)*.

### Argentina

Like other South American countries, Argentina does not offer exclusion testing. It is free of FMD without vaccination in Patagonia, the southern region of the country, and with vaccination in the rest of the country, where only cattle are vaccinated. The susceptible population includes 55 million cattle, 12 million sheep, 4 million goats, and 5 million pigs. The last FMD outbreak occurred in April 2006. Notification of the disease, including suspicious cases, is mandatory. In the event of a FMD incident, or even when it is only a suspicion, farmers and private veterinarians must immediately make a formal report to the local veterinary services. The latter are solely responsible for collecting and sending the samples to the NRL, which is the only laboratory authorized and accredited to perform the diagnostic tests with the appropriate biosafety and issue the final diagnosis. The costs are entirely covered by the national veterinary services.

Although private veterinarians or farmers should not collect and send samples of suspect cases, their participation and contribution to FMD surveillance is very significant. In fact, they receive training from the national veterinary services to recognize an FMD suspicion. Furthermore, farmers are associated to a local animal health association supervised by the National Food Safety and Quality Service SENASA. This association performs activities such as animal surveys, vaccinations, and surveillance. Apart from the concerns listed above, it is important to mention that the countries importing meat from Argentina (including the EU, USA, and China) regularly review the records of all suspect cases of FMD and would probably not accept an exclusion testing scheme instead of the current system.

## Discussion

Non-discriminatory FMD exclusion testing can help to quickly detect an introduction of this devastating disease into a previously free area, which is essential for its effective control. In countries where they are available, exclusion testing schemes are gaining acceptance among veterinary practitioners, but more needs to be done to promote the programs and increase awareness about foreign animal diseases in general.

At the same time, the possible pitfalls of exclusion testing should be kept in mind. Concerns about delays in the implementation of control measures are a major reason that many countries do not allow practitioners to submit samples for FMD exclusion. In these countries, any suspicion of FMD must be handled by the veterinary authorities, who will then take measures (quarantines, laboratory testing etc.) based on a risk analysis. The response to suspicions of FMD or other exotic animal diseases is often seen as an inalienable state activity to be carried out by the national animal health service, leading to strong resistance to the concept of non-discriminatory exclusion testing in the hands of private practitioners and laboratories other than the central NRL.

In this context it is important to emphasize that exclusion testing may complement, but cannot replace formally declared suspicions! A lot of the disagreement about non-discriminatory exclusion testing comes from the fact that in many countries, as soon as FMD is even *considered* in the differential diagnosis of a clinical case, it must be reported to the authorities. By contrast, other countries only require the notification of an actual *suspicion*. Where the lines between “no suspicion,” “weak suspicion” (consideration as a differential diagnosis) and “well-founded suspicion” are to be drawn is critical but often left to the individual private practitioner or government official.

It is clear, however, that a key step for the success of non-discriminatory FMD exclusion testing is the decision made by the private veterinarian during the farm visit; i.e., whether they consider FMD likely, unlikely or even rule it out completely (as depicted in Figure 2B). Therefore, any country considering the implementation of an exclusion testing scheme should establish guidelines to align the criteria of the private veterinarians with the officially desired benchmark. If the clinical and epidemiological picture clearly warrants directly raising a suspicion with the veterinary authorities, this must be done immediately in order to quarantine the infected farm and ensure prompt laboratory diagnosis.

Where decentralized FMD testing is available, it is critical that all laboratories involved operate to the highest diagnostic standards, particularly when negative results obtained in a regional laboratory are not sent to the NRL for confirmation. Moreover, the official veterinary system should be aware in real time of any ongoing non-discriminatory exclusion testing to keep records and to be alert in case a positive result appears. In addition to high sensitivity, diagnostic pipelines used for exclusion testing must have very high specificity to avoid false-positive results. False-negative results can obviously be catastrophic, but also the potential for false positives reduces the enthusiasm of farmers, veterinarians and government officials to endorse exclusion testing schemes. Due to the infrequent nature of FMD incursions into free countries and the multiplicity of factors that can affect FMD recognition, it is difficult to obtain empirical evidence for the effectiveness of any particular detection measure, including the relatively new exclusion testing schemes.

Either way, in order to maintain the freedom from FMD in any country, active and informed participation at all levels—professional farmers and hobbyists, practitioners, educators and veterinary services—is essential. The longer a disease has not occurred in a country, the more important it is to make sure that all stakeholders are aware of the risk of reintroduction, are well-equipped to identify its clinical signs and know what steps to take should the occasion arise. Online resources (such as webinars or phone apps with visual references) can be of great utility in the implementation of non-discriminatory exclusion testing schemes. In addition, farmers and practitioners must have confidence in the official animal health authorities, and must be assured that any notification is immediately responded to, a quick diagnosis is obtained, and preventive regulatory measures are applied for as short as possible. Non-discriminatory exclusion testing is one of a range of measures that can be considered to improve the detection of FMD incursions.

## Author Contributions

MB had the idea for this article. ME wrote the original draft. All authors revised and expanded the original draft and approved the submitted manuscript.

## Conflict of Interest

The authors declare that the research was conducted in the absence of any commercial or financial relationships that could be construed as a potential conflict of interest. The handling editor declared a shared affiliation, though no other collaboration, with one of the authors (DO'B) at the time of the review. The handling editor declared a past co-authorship with several of the authors (ME, CN, and DK).

## Footnotes

^1^MKS-Verordnung in der Fassung der Bekanntmachung vom 18. Juli 2017 (BGBl. I S. 2666, 3245, 3526).

^2^Untersuchungen zum Zwecke des Ausschlusses von Tierseuchen. Erlass BMASGK-74730/0002-IX/B/10/2019.

^3^United States Code, 2006 Edition, Supplement 4, Title 21, Chapter 4, Subchapter III – Prevention of Introduction and Spread of Contagion, Sec. 113a – Establishment of research laboratories for foot-and-mouth disease and other animal diseases. Pub. L.116-141; 2020-06-03.
